# Sensing Enzyme Activation Heat Capacity at the Single-Molecule Level Using Gold-Nanorod-Based Optical Whispering Gallery Modes

**DOI:** 10.1021/acsanm.1c00176

**Published:** 2021-03-29

**Authors:** Sivaraman Subramanian, Hannah B.L. Jones, Simona Frustaci, Samuel Winter, Marc W. van der Kamp, Vickery L. Arcus, Christopher R. Pudney, Frank Vollmer

**Affiliations:** † Living Systems Institute, Department of Physics & Astronomy, University of Exeter, Exeter EX4 4QD, U.K.; ‡ Department of Biology and Biochemistry, Centre for Biosensors, Bioelectronics and Biodevices, University of Bath, Bath BA2 7AY, U.K.; § School of Biochemistry, University of Bristol, Bristol BS8 1TD, U.K.; ∥ Te Aka Ma̅tuatua - School of Science, University of Waikato, Hamilton 3240, New Zealand

**Keywords:** single molecules, whispering gallery modes, plasmonics, biosensing, catalysis, enzyme mechanism, the heat capacity of catalysis

## Abstract

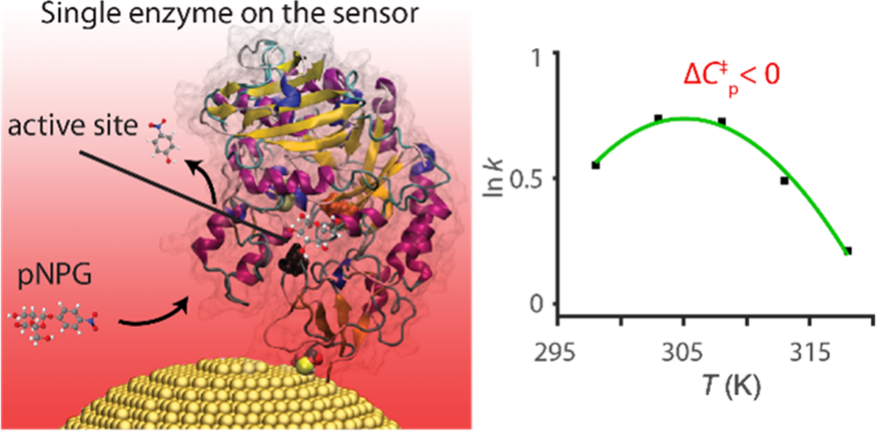

Here, we report a label-free gold nanoparticle-based single-molecule optical platform to study the immobilization, activity, and thermodynamics of single enzymes. The sensor uses plasmonic gold nanoparticles coupled to optical whispering gallery modes (WGMs) to probe enzyme conformational dynamics during turnover at a microsecond time resolution. Using a glucosidase enzyme as the model system, we explore the temperature dependence of the enzyme turnover at the single-molecule (SM) level. A recent physical model for understanding enzyme temperature dependencies (macromolecular rate theory; MMRT) has emerged as a powerful tool to study the relationship between enzyme turnover and thermodynamics. Using WGMs, SM enzyme measurements enable us to accurately track turnover as a function of conformational changes and therefore to quantitatively probe the key feature of the MMRT model, the activation heat capacity, at the ultimate level of SM. Our data shows that WGMs are extraordinarily sensitive to protein conformational change and can discern both multiple steps with turnover as well as microscopic conformational substates within those steps. The temperature dependence studies show that the MMRT model can be applied to a range of steps within turnover at the SM scale that is associated with conformational change. Our study validates the notion that MMRT captures differences in dynamics between states. The WGM sensors provide a platform for the quantitative analysis of SM activation heat capacity, applying MMRT to the label-free sensing of microsecond substates of active enzymes.

## Introduction

It is increasingly apparent that to fully understand enzyme activity, one must take account of the protein intramolecular dynamics and fluctuations on a range of time and length scales.^1^ While intramolecular dynamics are observable via a range of approaches, linking the dynamics to catalytic activity is extremely challenging (and controversial). Enzyme/protein dynamics have the potential to affect a range of processes from substrate binding, gating of electron transfer, and conformational sampling of different reactive complex geometries and potentially even affecting catalysis.

The temperature dependence of enzyme turnover is used as a window into the fundamental thermodynamics and processes involved in catalysis. These data are often extracted by fitting kinetic data to the Eyring equation, giving an activation enthalpy and entropy. Recently, however, a large body of data has emerged where enzymes do not conform to these models. Hobbs *et al.* first proposed a new model for interpreting these data called macromolecular rate theory (MMRT).^2,3^ This model postulates an activation heat capacity (Δ*C*_*P*_^‡^) associated with the change in conformational dynamics (along the reaction coordinate) between the enzyme–substrate complex and the enzyme–transition state complex,
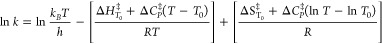
1where Δ*H*^‡^ is the change in enthalpy and Δ*S*^‡^ is the change in entropy between the ground and transition state of the reaction at an arbitrary reference temperature (*T_0_*).

We have previously shown that maltose-inducible α-glucosidase (MalL) deviates from Eyring/Arrhenius kinetics in the temperature dependence of the rate of hydrolysis of the substrate 4-nitrophenyl-β-d-glucopyranoside (pNPG) into a β-d-glucose and *p*-nitrophenol, manifesting as a significant negative Δ*C*_*P*_^‡^.^2,4^ We have conducted extensive molecular dynamics simulations for MalL in both the enzyme–substrate complex and a pseudo-enzyme–transition state complex (5 μs for each), which identifies the source of this negative Δ*C*_*P*_^‡^ as arising from the difference in conformational fluctuations between the ground and transition state as per the MMRT model.^5^ These changes in fluctuations are distributed across the enzyme domains and thus account for the large negative values of the activation heat capacity (approximately −5.9 kJ mol^–1^ K^–1^)^4^ and the pronounced deviations (curvature) in Eyring/Arrhenius plots.

Ideally, one would like to track the MalL enzyme conformational dynamics associated with turnover in experiment, as well as separate the contributions from multiple reactive conformations (MRCs) if present. Single-molecule (SM) studies excel at exposing rare or transient states and disambiguating the presence of several different conformational states within an equilibrium. Most SM enzyme kinetic studies are based on the detection of turnover via the change in fluorescence of a substrate/product, a cofactor, or a FRET probe.^6^ These fluorescence SM studies provide detailed information on conformational dynamics associated with turnover by monitoring chemical reactivity after labeling of the protein with the fluorophores. The FRET donor/acceptor probes have been employed to track conformational dynamics at the SM level. The resolution of this approach has been particularly successful in studies of large-scale protein conformational changes (∼10 Å).^7^

**Scheme 1:**
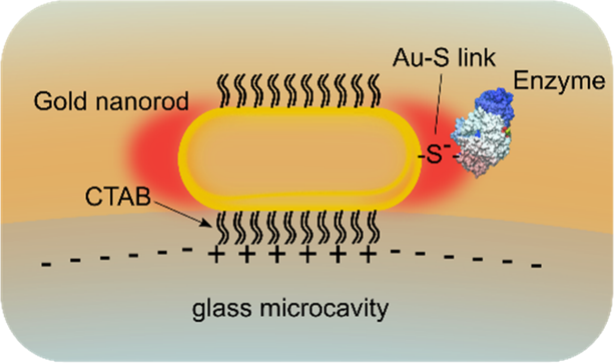
Schematic of the Gold Nanorod-Based Optoplasmonic Sensor Assembly with an Enzyme Attached via Au–S Linkage

Here, we present a nanosensing approach based on optical whispering gallery modes (WGMs) enhanced by gold nanoparticles that can complement these studies and probe the MalL conformational dynamics at the SM level. The strong decay of the evanescent field of gold nanoparticles within the size of a single protein (∼6 nm decay length) is particularly suitable to capture the small scale conformational fluctuations of MalL that are functionally relevant.^5^ The nanosensor system in combination with the lock-in technique applied in this work enables the real-time tracking of small conformation fluctuations of an enzyme on the sub-ms time scale and without the use of fluorophores. This single-molecule study of enzyme activity at a biosensor interface adds a label-free (non-fluorescent), rapid (μs), and highly sensitive approach to the range of available fluorescence-based techniques that are critically important to study enzyme dynamics at an interface.^8^ We have recently reported a single-molecule optical sensor that utilizes plasmonic gold nanoparticles to monitor the interaction, immobilization, and activity of single enzymes on gold nanoparticles coupled to optical microcavities.^9,10^ Using this sensor, we have been able to track the conformational movements of a DNA-polymerase^10^ and surface reactions of small 100 Da molecules.^11^ Here, we extend the previous approach by improving the time resolution of the system to microseconds and immobilizing the enzyme in a specific manner using a surface-exposed thiol group. Scheme 1 shows the sensor assembly. The sensor is composed of positively charged plasmonic gold nanorods electrostatically attached to a fused silica microsphere (diameter ≈ 80 μm). The MalL enzymes are then attached to the gold nanosensors using Au–S linage via a solvent-exposed cysteine group.

**Figure 1:**
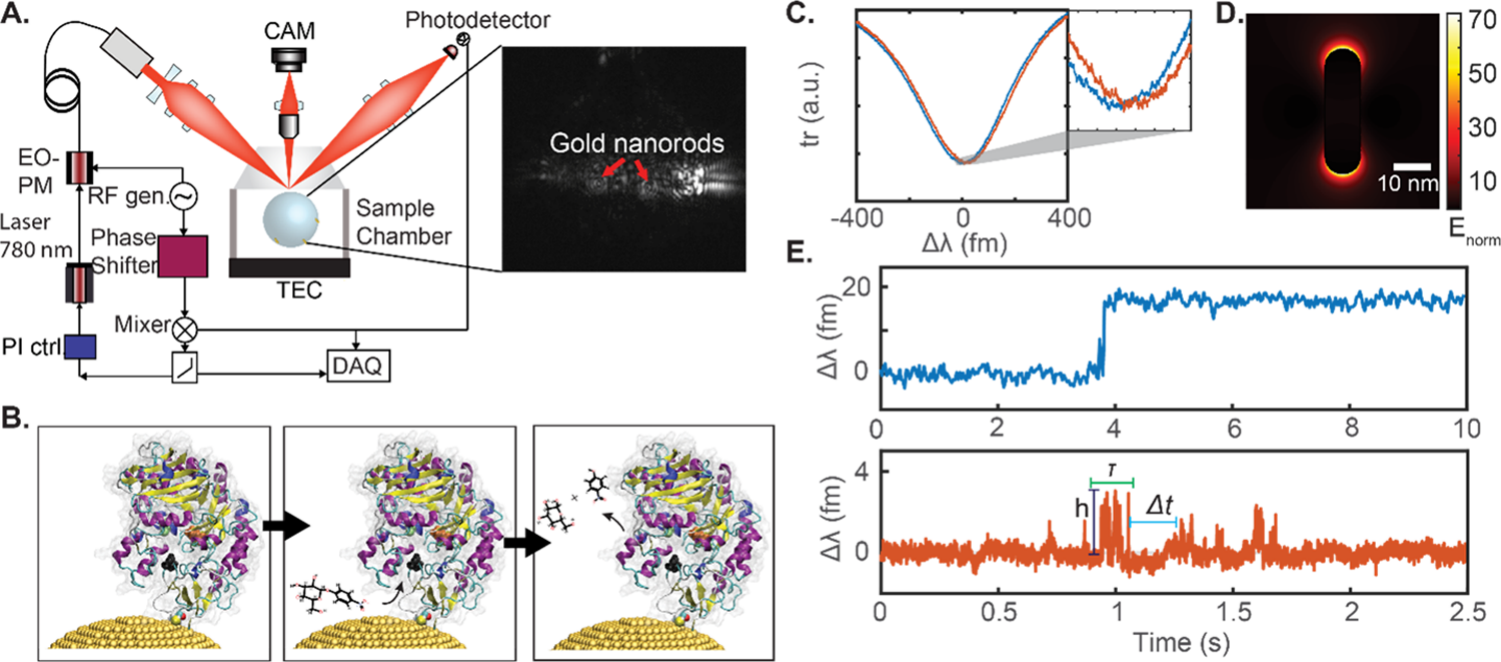
(A) Schematic of the experimental setup. Zoom-in shows a photograph of the sensor, that is, a spherical WGM resonator with multiple gold nanorods attached to its surface. (B) Illustration of the turnover of an enzyme attached to the ends of the gold nanorods on the WGM resonator surface. (C) WGM transmission spectra before (blue) and after (orange) enzyme binding to gold nanoparticles. The inset shows the zoom-in of the transmission spectrum. (D) Calculated electric field distribution around a plasmonic gold nanorod of size 10 × 35 nm using a boundary element method (BEM) via the MNPBEM toolbox.^12^ (E) Time traces of the WGM resonance wavelength Δλ for the binding of single enzymes (top) and the turnover of the substrate by the immobilized enzyme (bottom). Various aspects of the signal such as event amplitude (*h*), event/interaction dwell time (τ), and the time between consecutive events (Δ*t*) are highlighted for reference.

Figure 1A shows the experimental setup used to excite the optical WGM within ∼80 μm glass microspheres. The conformational changes of the enzyme on the gold nanoparticles (Figure 1B) then translate into shifts of the WGM resonance position (Figure 1C). The different overlaps of the plasmonic near-field (Figure 1D) with the enzymes undergoing conformational changes result in a different volume integral leading to the WGM shift signals as previously reported.^10^ To benefit the readers’ understanding of our data, Figure 1E plots the time traces of the WGM resonance shifts for the binding of a single enzyme (top) and the turnover of the substrate by the immobilized enzyme (bottom). The step-like transition in the signal indicates the binding of single enzymes to the gold nanorod. The spike transitions in the signal indicate turnover events. Various aspects of the signal such as the event amplitude (*h*), event/interaction dwell time (τ), and the time between consecutive events (Δ*t*) are estimated from the time traces. These data then reflect the rate and magnitude of conformational changes detected at the SM level by the WGM shifts.

Herein, we show that WGMs can disambiguate the catalytic rates associated with multiple reactive conformational substates (MRCs) during enzyme catalysis. By monitoring conformational changes, we can uncover the presence of putative conformational sampling of “reaction-ready” conformations. Finally, we show that the temperature dependence of the enzymatic rates shows significant curvature, even where we can exclude the contributions from MRCs. These data provide powerful validation of the MMRT model capturing differences in dynamics between states and point the way forward for understanding the role of conformational substates and dynamical fluctuations in enzyme catalysis. Moreover, our data begin to uncover the lower sensitivity bound of WGMs since we can correlate to extensive molecular dynamics simulations.^5^

## Results and Discussion

Establishing and validating the SM setup. Figure 1A shows the schematic of the experimental setup used for studying single enzymes. A spherical glass resonator of a diameter of ∼80 μm fabricated at the tip of a single-mode optical fiber acts as the WGM microcavity. Plasmonic gold nanorods (A12-10-750-CTAB, Nanopartz Inc., USA) are attached to the WGM cavity as previously reported.^9^ The total number of gold nanorods attached to the WGM cavity surface is monitored (Supporting Information, section S1). Either laser scanning or the error signal of a Pound–Drever–Hall (PDH) lock (more details in the Supporting Information, section S2) obtains the WGM spectral information and WGM shift signals. Further information on the classification of the spike-like transitions to “events” and the data processing are provided in the Supporting Information, section S3.

We have generated a variant of MalL (S135C MalL) for attachment to the gold nanorods used in the SM studies via a thiol linkage to the introduced cysteine. The cysteine is located in a loop region on the surface of the protein, distal from the active site. Figure S4 shows the steady-state kinetics of S135C MalL and the temperature dependence of the saturated steady-state rate constant. We find that S135C MalL is catalytically active in solution with a *k*_cat_ that is smaller than that for the wild-type enzyme (∼5 times, potentially due to intermolecular disulfide bond formation). From Figure S4, both the *K*_M_ and Δ*C*_*P*_^‡^ values are similar between WT MalL and S135C MalL measured across a similar temperature range, where *K*_M_ = 107.9 ± 34.2 and 141.2 ± 39.6 μM, and Δ*C*_*P*_^‡^ = −4.5 ± 0.4 and – 5.0 ± 0.6 kJ mol^–1^ K^–1^, respectively. That is, S135C MalL is kinetically similar to WT MalL at least at the level of detection provided by ensemble steady-state kinetics. We note that we are unable to extract reliable ensemble steady-state kinetic data for nanorod-bound S135C MalL owing to the extremely poor solubility of the enzyme–nanorod complex in solution (<1 nM due to agglomeration), meaning that the enzyme degrades before an observable absorption change can be monitored. However, we demonstrate turnover in our SM assays.

Assembly of the sensor is as we have reported previously.^10^ The detailed description of the sensor assembly is described in the Supporting Information, section S1. After sensor assembly, single enzymes are immobilized on the gold nanorod surface. On the addition of the enzyme, a step-change in the WGM resonance (as illustrated in Figure 1D) indicates a permanent binding of S135C MalL to the gold nanorod, whereas a series of spikes indicate a transient interaction of the enzyme with the nanorod.

**Figure 2:**
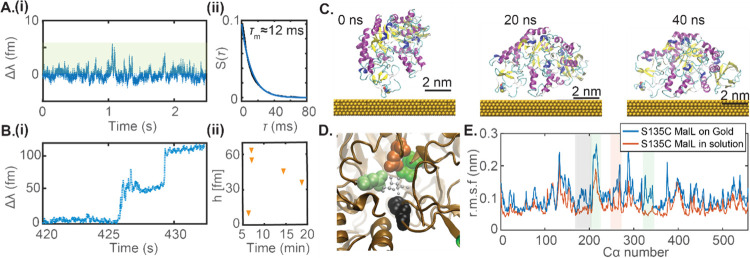
(A) (i) Sensor signals due to transient interaction of the S135C MalL enzyme with the gold nanorod in the absence of the reducing agent. The signals in the green shaded region are detected as peaks. (ii) Survivor function and corresponding exponential fit to the dwell time of spike events. (B) (i) Binding of the enzyme to the ends of gold nanorods in the presence of a reducing agent. (ii) Binding step height over time. The plot shows a total of five binding events observed during a 20 min measurement interval. Experiments were performed in 20 mM HEPES buffer, pH ≈ 7 at ∼298 K. (C) Snapshots of S135C MalL on a gold surface at 20 ns time intervals obtained from MD simulations. Representations rendered using VMD.^13,14^ (D) A zoom-in of the active site of S135C MalL. The glucose binding residues F144 (black), D199 (green), E255 (orange), and D332 (green) are represented as solid spheres, and the d-glucose substrate is represented as ball-and-stick (grey). (E) Root-mean-square fluctuation of the C-alpha atoms of S135C MalL free in solution (orange) and S135C MalL on a gold surface (blue). The shaded regions mark the C-alpha atoms around the active site residues. Simulations were performed in explicit water at 300 K.

Figure 2A,i shows the WGM signals obtained by the PDH error signal upon the addition of ∼100 nM of S135C MalL to the sample chamber. The spike-like transient signals obtained indicate the interaction of the enzyme with the gold nanorod surface but not formal binding. The inability of the enzyme to bind is likely due to the unavailability of free solvent-exposed thiols to bind to the gold nanoparticle surface. This indicates a possible oxidation of the thiol or a dimerization of the enzymes via disulfide bonds. Figure 2A,ii shows a plot of the survivor function *S*(τ) of the interaction dwell times τ. Here, the survivor function is defined as, *S*(τ) = *P*(*T* > τ), where *P*(τ) is the probability function and *T* is a continuous random variable in time. A single exponential fit to S(τ) provides a mean interaction dwell time of τ_m_ ≈ 12 ms. The value of τ_m_ is in the order of the scanning time of 20 ms typically used in WGM single-molecule sensing by laser scanning approaches.^10^ Hence, the PDH lock-in technique developed in this work and its microsecond time resolution is required to resolve these fast single-molecule events. Moreover, the relatively large (ms) dwell time indicates the presence of electrostatic interactions between the enzyme S135C MalL and the gold nanorod surface. Previously, Kim *et al*. showed that the interaction of freely diffusing DNA polymerase with gold nanorods could not be detected with a similar sensor.^10^ Using Monte Carlo simulations, they estimated that DNA polymerase molecules would diffuse away from the evanescent field of the gold nanorods within a few microseconds assuming no electrostatic interactions between the molecule and nanorod surface (see Supporting Information, section S9, Kim *et al.*(10)).

To remove any dimer formation before attachment to the nanorods, the reducing agent Tris(2-carboxyethyl)phosphine hydrochloride (TCEP-HCl) was added to the buffer. Figure 2B,i shows the step changes in WGM resonance obtained upon the addition of TCEP-HCl (control experiments show that TCEP-HCl itself does not produce any measurable sensor signals). These steps indicate a permanent binding of S135C MalL to the nanorod surface. Figure 2B,ii shows the step heights measured over 20 min. We observe five enzyme binding steps that contribute to signals of enzyme–substrate interactions in the next set of experiments. Steps in WGM linewidth may also be observed due to the shifting of unresolved split-modes as reported previously.^15^ However, the quality factor of the resonance does not reduce significantly upon enzyme immobilization due to the low losses induced by the enzymes. We note that the covalent binding of the enzyme to the nanorods restricts diffusive motion during turnover. The close separation of the enzyme and nanorod ensures very high sensitivity for WGM-based detection of conformational fluctuation.

We have performed molecular dynamics (MD) simulations to study the impact of the gold surface on the intramolecular dynamics of S135C MalL. MD simulations were performed in the GROMACS software package^16^ for both the free enzyme and enzyme near a Au <111> surface. A 40 ns simulation was performed for both cases (see Methods). Figure 2C shows the enzyme S135C MalL after 0, 20, and 40 ns of MD simulation (refer to the Supporting Information, section S5, for more details on the simulation setup). The simulations show that the enzyme orients in a specific way and spreads on the Au surface. Several residues of S135C MalL were identified to interact with the gold surface, including Lys, Pro, Asp, Cys, Tyr, and Glu (see Figure S6A). From our simulations, the choice of the mutation site ensures that the active site of the enzyme is situated 2–3 nm away from the surface of the gold nanorod where the WGM signal enhancement by the nanorod is still approximately 20-fold (see Figure S6B). Figure 2D shows a zoom-in of the active site and Figure 2E plots the root-mean-square fluctuations (r.m.s.f) of the S135C MalL free in solution and on a Au <111> surface. The r.m.s fluctuations of the C-alpha atoms near the active site increases when the enzyme is interacting with a Au surface (Figure 2E). We are cautious in over-interpreting this finding from a single trajectory, but we note that Hobbs *et al.* studied the impact of mutations on the wild-type enzyme WT MalL and observed that an increase in flexibility of the atoms near the active site caused a decreased catalytic rate.^2^ This would then track well with the decreased catalytic rate observed for S135C MalL in our SM experiments discussed below.

### Sensitivity of the WGMs to MalL Conformational Fluctuations Associated with Enzyme Turnover

**Figure 3:**
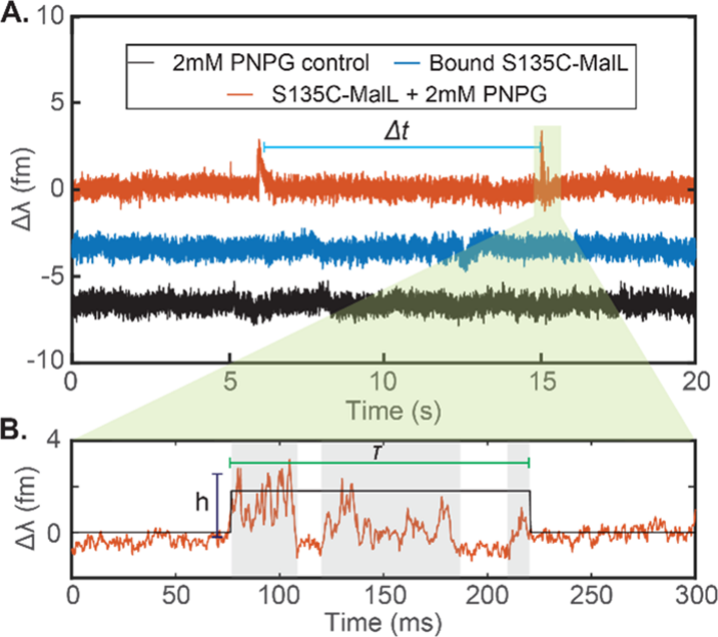
Measurement of enzyme dynamics. (A) Plots of the sensor signals vs time with the substrate present and in the absence of the enzyme (black), with the enzyme present (blue), and with the enzyme and substrate present (orange). (B) Zoom-in on a 300 ms section of (A) (orange trace). The spike-like events can occasionally (approximately 10–20% of traces) be discerned as multiple discrete events.

After immobilization of the enzyme, the excess free enzyme in the solution is removed by rinsing the sample chamber with buffer. Then, the signals from the enzyme–substrate interactions are monitored via the PDH error signal. Figure 3A plots examples of signals obtained at various steps of the experiment. The signals in Figure 3A are downsampled to 2.5 kHz and corrected for drift to improve the presentation (see the Supporting Information, section S3). The signal peaks above the noise level are grouped into single-molecule “events” based on an algorithm described in the Supporting Information, section S3. The noise level of the signal is measured as 3σ, where σ ≈ 0.4 fm is the standard deviation of the background. Before the immobilization of the enzyme, no discernible peaks above the 3σ noise are found even in the presence of 2 mM pNPG substrate (black trace). These data show that the substrate does not interact with the gold nanoparticle measurably and so our SM data are not convolved of interactions of the substrate with the nanorod. Moreover, after enzyme immobilization, no spike events are observed, indicating that any diffusive movements of the enzyme on the gold surface are restricted. That is, our data demonstrate that the detected signals are associated with turnover.

**Scheme 2:**
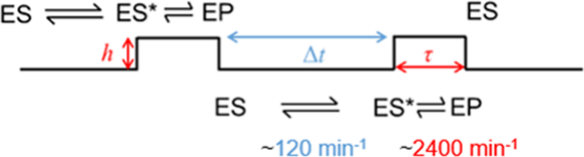
Proposed Mechanistic Scheme Relating to SM Data of S135C MalL in the Presence of pNPG

Upon addition of 2 mM pNPG, spikes above the noise are observed (Figure 3A; orange trace). Figure 3B shows a 300 ms zoom-in of the signals measured in the presence of an enzyme and substrate. At the single-molecule level, the data are not convolved of forward/reverse steps as in ensemble kinetics. That is, the observation of step-like behavior in SM traces is commensurate with a single process and the concept of “saturation” of the kinetics is not meaningful at the SM level. We anticipate substrate binding to be fast, and we do not find evidence from our previous extensive MD simulations for a conformational change associated with substrate binding larger than the changes observed in the substrate-bound state.^5^ Therefore, we anticipate the observed step-like behavior in the transients reflects conformational change/fluctuation associated with enzyme turnover. That is, for further analysis, the SM traces are broken into mechanistic steps as shown in Scheme 2.

The traces comprise four chemically distinct processes putatively: (i) rapid binding of substrate to give ES; (ii) conformational fluctuation/rearrangement to form the catalytically competent binary complex, ES* (a transition state-like conformation), reflected in Δ*t*; (iii) decay of this complex and chemical turnover to form product EP, which is reflected in τ; and (iv) product release and new substrate binding.

The time scale of Δ*t* and τ are an order of magnitude different (approximately 120–2400 min^–1^, see the Supporting Information, section S3), and so turnover would be rate-limited by the process reflected by Δ*t*, the supposed timescale for rearrangement to a catalytically competent binary complex. Indeed, the timescale of Δ*t* from the single-molecule experiment is of a similar order of magnitude as for S135C MalL measured via ensemble kinetics (Figure S5) being ∼120 and 280 min^–1^. Our data suggest that the S135C MalL turnover is slower than WT MalL at the ensemble level as the amino acid substitution has slowed the formation of ES* to be kinetically observable. We consider the extracted kinetic data in more detail below.

### Temperature Dependence of Enzymatic Rates

**Figure 4:**
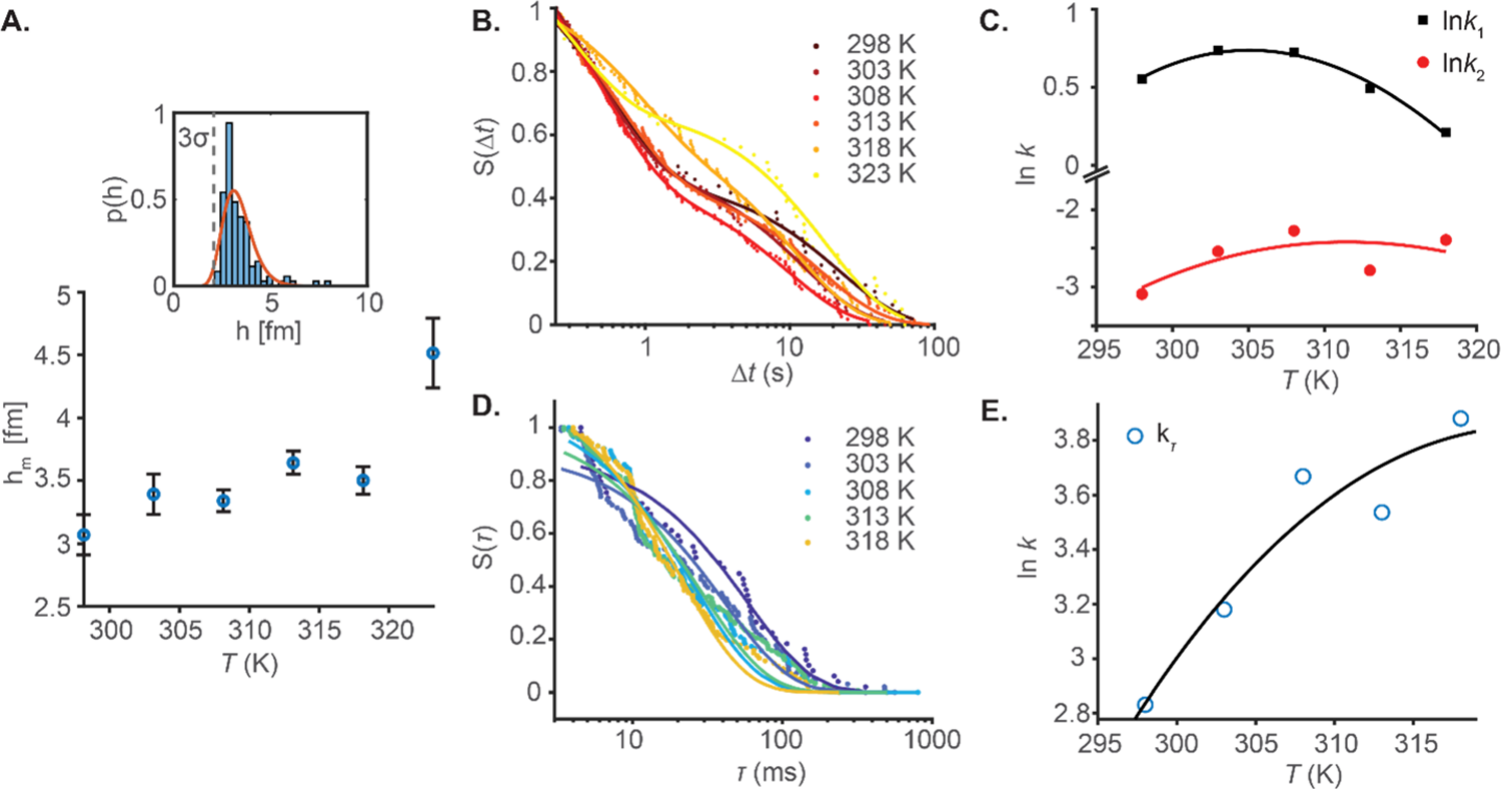
Temperature dependence of enzymatic rates. (A) Mean event amplitudes *h_m_* over temperature. The mean event amplitudes are estimated from log-normal fits to the distribution of event amplitudes as shown in the inset. Data in the inset were measured at 308 K. (B) Temperature dependence of Δ*t* distributions. Solid lines are the fit to eq 2. (C) ln*k* values extracted from panel (B). Solid lines are the fit to eq 1. (D) Temperature dependence of τ distributions. Solid lines are the fit to eq 2 for a single exponential. Data at 323 K are omitted as the enzyme is likely denatured at this temperature. (E) ln*k* values extracted from panel (D). Solid line is the fit to eq 1. The temperature dependence experiments were performed in a 20 mM HEPES buffer and a pH of ∼7 with a substrate concentration of 250 μM.

Our SM data allows us to extract the temperature dependence of the rates for both the process reflected by Δ*t* and τ. Figure 4A shows the extracted mean *h* values reflecting the substrate binding and the associated conformational change as described in Figure 1. The mean *h* values are estimated from a log-normal fit to the distribution of event amplitudes as shown in the inset. Broadly, as the magnitude of *h* increases, the data reflect a more significant conformational change/fluctuation since the mean contribution from substrate binding should be constant. There is an evident trend of an increase in the value of *h* with temperature as one might expect for the increasing molecular flexibility associated with increasing temperature. Indeed, we have previously shown that this is the case with both WT MalL and a mutant V200S MalL using red-edge excitation shift (REES) spectroscopy.^4^ Below 323 K, the data have a weak upward trend and the data between 298 and 318 K are the same within error. At 323 K, the *h* value increases dramatically and likely reflects protein unfolding. Indeed, at 323 K, we find that there are hardly any events to capture, which is consistent with our suggestion that the enzyme is only stable up to 318 K for the length of our assay. We, therefore, do not consider the kinetic data arising from this temperature. That the *h* data between 298 and 318 K are the same within error suggests that the protein is structurally similar and not unfolding at these temperatures.

Having established that unfolding is not affecting the kinetic data in our SM traces, we extract the kinetic data from the distribution of arrival times, Δ*t*, as Scheme 2 (Figure 4B). We find that at all temperatures studied, the data can be adequately fit with a function comprising two exponentials,
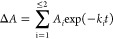
2where *A* is the amplitude, *k* is the observed rate constant (*k*_obs_) for the *i*^th^ exponential component (up to two), and Δ*A* is the total amplitude change. We find that the individual exponentials have similar amplitudes, comprising ∼60 and 40% of the total amplitude for *A*_1_ and *A*_2_, respectively, at each temperature. That the data are multiphasic argues that at least two separate states can be resolved from the single-molecule data. Given that the Δ*t* data putatively reflect the formation of a reaction competent geometry from an initial ES complex (as Scheme 2), we suggest these data are exposing different ES complex geometries. These geometries then ultimately decay to a structurally similar (not necessarily identical) ES* complex. More than two states might be present since the exponential fitting is only able to reliably resolve rates that are ∼1 order of magnitude apart (as is the case for these data). Nevertheless, the data suggest an equilibrium of ES complex conformations and is consistent with the vast bulk of findings from single-molecule enzyme studies.^17−23^

Figure 4C shows the temperature dependence of the extracted rates from Figure 4B for the kinetic rates from each phase. The solid lines show the fit to eq 1, and the resulting parameters are given in Table 1. From Table 1, we find, as with WT MalL at the ensemble level, that the data show significant plot curvature. The temperature plots give negative Δ*C*_*P*_^‡^ values for both *k*_1_ and *k*_2_, that is, −5.5 ± 5.1 and – 2.2 ± 3.4 kJ mol^–1^ K^–1^, respectively, acknowledging the relatively large attendant error. A consequence of the plot curvature is that the temperature where the theoretical maximum rate occurs (*T*_opt_) can be extracted. From Table 1, the magnitude of *T*_opt_ is ∼5 K different for *k*_1_ and *k*_2_ and with an order magnitude difference in the rate at *T*_opt_. For a fraction of the events reflected by τ (10–20% depending on temperature), we find that the traces can be decomposed into three discrete subdomains (shaded regions in Figure 3B). See the Supporting Information, section S3, for peak detection and event classification details. Potentially, these data reflect the observation of discrete states with the conformational equilibrium suggested by the multiphasic kinetics shown in Figure 4B and as described above. Figure 4D shows the fits of a single exponential function to the kinetic data extracted for τ, which we suggest reflects the rate of decay of the reactive ready complex (ES*) and chemical turnover as in Scheme 2. We note that one can fit a sum of exponential functions to these data and significantly improve the fitting statistics, but the fits generally remain poor with either two or three additional exponentials (eq 2). Fitting multiple exponentials to rate data typically only performs well where there is a ∼1 order magnitude difference between exponential phases, which does not appear to be the case here. The single exponential fits, therefore, provide a useful means to interpret the data and we feel is the most robust analytical approach given the complexity of the data set.

**Table 1:** Extracted Parameters from Fitting Data in Figure 4 to Eq 1

	Δ*t*		τ
	*k*_1_	*k*_2_	*k*_τ_
*T*_opt_ (K)	305	312	320
*k*@*T*_opt_ (s^–1^)	2	0.08	47
Δ*H*^‡^ (kJ mol^–1^)^a^	–14 ± 15	10 ± 10	38 ± 11
Δ*S*^‡^ (kJ mol^–1^ K^–1^)^a^	1.02 ± 0.05	1.06 ± 0.03	1.15 ± 0.04
Δ*C*_*P*_^‡^ (kJ mol^–1^ K^–1^)	–6 ± 5	–2 ± 3	–3 ± 3

a data at 307 K.

The temperature dependence of the resultant τ rate data is shown in Figure 4E, with the solid line being the fit to eq 1 and the resulting parameters given in Table 1. These data show evident curvature, similar to steady-state measurements over the same temperature range (Figure S4), with Δ*C*_*P*_^‡^ = −2.9 ± 2.6 kJ mol^–1^ K^–1^. That is, for the putative chemical step, disambiguated from other rate-limiting steps, we find a similar large negative curvature as observed for the wild-type enzyme-free in solution. Moreover, we note that the extracted *T*_opt_ and *k* values are similar to the wild type enzyme in solution, where we have suggested that steady-state turnover (unlike the variant enzyme used here) is rate limited by chemical turnover.

## Discussions and Conclusions

To date, all MMRT data has arisen from ensemble enzyme kinetic measurements. Ideally, one wishes to capture kinetic data for a single chemical step and in the absence of interconverting conformational substates that are differently reactive (different thermodynamic barriers). The convolution of different steps and microscopic substates conceivably could give rise to the kind of curvature in temperature dependence plots that are fit using MMRT. Given the extreme sensitivity of the plasmon-enhanced WGM sensor, this SM approach is uniquely capable to discern the temperature dependence of truly discrete kinetic steps and conformational substates. The WGM-plasmonic sensor presented here has the potential to capture extraordinarily small conformational changes. Our previous work has validated the use of this sensor to capture enzyme turnover associated with conformational changes that are relatively large scale (∼10 Å).^10^ In the present case of MalL, our previous extensive molecular dynamics simulations allow us to infer that the sensitivity of the WGM-plasmonic sensor is in the low Å range since MalL does not undergo any large scale conformational changes. Our data, therefore, illustrate the extraordinary sensitivity of this sensor and the enormous potential in tracking functionally relevant conformational change for a large range of proteins.

**Scheme 3:**
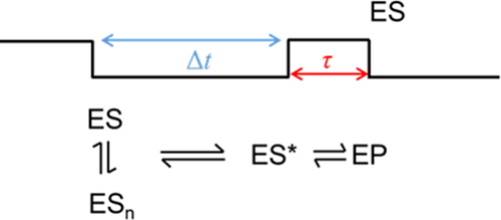
Proposed Mechanistic Scheme Relating to SM Data of S135C MalL Turnover Based on Data from Figure 4

From MD simulations, we find that binding of the enzyme to the Au surface does affect the native enzyme dynamics and this correlates with a decrease in the rate of turnover. Serendipitously, these changes, and the sensitivity of the WGMs, allow us to uncover multiple steps during MalL turnover, which would otherwise not be resolvable. These turnover-associated conformational changes can be assigned to putative steps during MalL turnover based on a minimal kinetic scheme similar to Scheme 2. The power of SM approaches allows us to further interrogate substates within each step. Our data point to the presence of multiple reactive substates within each discernable step. That is, as shown in Scheme 3, our data suggest an equilibrium of ES complex geometries, which can interconvert between the related equilibrium of reactive conformations (ES*).

We have presented a nanosensor system based on plasmonic gold nanoparticles that is capable of extracting single enzyme kinetics with a sub-ms time resolution without the use of fluorescent labels. This sensor system allows for the accurate control of temperature and hence investigates the temperature dependence of not just individual steps but also the resolvable substates within steps. Using this nanosensor, we have studied a test enzyme MalL, which has emerged as the paradigmatic system for studying enzyme temperature dependencies and the MMRT physical mode. Our single molecule data show evident curvature in the temperature dependencies of each of the resolvable steps during MalL turnover as well as differences between substates associated with the formation of a putative reaction-ready conformation. That is, we find that a negative Δ*C*_*P*_^‡^ value is a consistent feature of individual steps even accounting for the presence of microscopic substates within those steps. Therefore, given that Δ*C*_*P*_^‡^ reflects differences in fluctuations between states, it is logical to speculate that there is a reduction in the conformational fluctuations from ES → ES* → TS (transition state).

Our study, therefore, provides some of the most compelling evidence to date for the MMRT model and its physical underpinning, which is rooted in differences in the distribution of states. Moreover, our data point to the power and utility of gold nanoparticle-based WGM sensors in studying enzyme turnover at a resolution that enables complex physical models to be understood.

## Methods

### Sample Preparation

Site-directed mutagenesis of MalL to generate S135C and S135C/V200S constructs was carried out by Genscript (Piscataway, NJ, USA). The expression and purification of full-length *Bacillus subtilis* 168 MalL were carried out as outlined previously.^2,4^ After purification, MalL was buffer exchanged into 20 mM HEPES 20 mM TCEP-HCl (Sigma Aldrich, 646547-10X1ML) pH 7.0 using spin concentration (10 kDa cut-off). Here, the protein was diluted at a 1:10 ratio and concentrated 10-fold for 6 times.

### Experimental Setup

An external cavity diode laser (Toptica TA pro 780HP, Toptica GmbH, Munich, Germany) is used as the excitation laser. Total internal reflection at an N-SF11 prism surface is used for efficient coupling to the WGMs. The WGM microcavity is an 80–90 μm diameter glass microsphere fabricated at the end of a single-mode optical fiber (SMF 28e, Corning GmbH, Germany). A 30 W CO2 laser, λ ≈ 10.6 μm (Synrad 48–2, Novanta Inc., WA, USA), is used at 10–15% peak power to melt the glass fiber into a sphere using a home-built setup. A v-shaped chamber cut from polydimethylsiloxane (PDMS) is used for sample loading.^24^ The sample volume was 300–400 μL in all experiments. cetrimonium bromide-capped gold nanorods 10 × 35 nm (A12–10-750-CTAB, Nanopartz Inc., USA) are used for the plasmonic enhancement of the WGM. The nanorods were attached to WGM microcavity at a pH of 1.7, and the number of nanorods attached is monitored in real-time. A thermoelectric element (32 W TEC, Thorlabs GmbH, Germany), driven by a PID temperature controller (5310 TEC Source, Arroyo Instruments LLC, USA), and a Pt-100 (362–9799, RS PRO, RS Components Ltd., UK) temperate sensor were used to control the chamber temperature.

### Molecular Dynamics Simulations

The MD simulations were performed in the GROMACS software package. Two simulations were performed, one for the S135C-MalL free in water as solvent (free enzyme) and another for S135CMalL close to a Au <111> surface (enzyme-surface). The crystal structure of MalL (PDB 4 M56)^2^ was modified to introduce a mutation at residue 135 converting an SER to a CYS residue. The resulting structure was used for further simulations. The free enzyme simulations were performed using the OPLS^25^ force field, and the enzyme-surface simulations were performed using the GolP-OPLS^26^ force field. The MD simulations were performed for both systems in an NVT ensemble at 300 K, with temperature regulated using a Nosé–Hoover thermostat.^27,28^ All bonds containing hydrogen were constrained using the LINCS algorithm,^29^ and a 2 fs time step was used. All simulations were performed for 40 ns. Further details of the simulation are provided in the Supporting Information, section S5.

## Supporting Information Available

Assembly of nanorods with WGM, data acquisition (PDH lock) and data analysis methods, colorimetric assays, and MD simulation setup (PDF)
