# Supplementary material for: Sensing Enzyme Activation Heat Capacity at the Single-Molecule Level Using Gold-Nanorod-Based Optical Whispering Gallery Modes
Source: ACS Appl Nano Mater. 2021 Mar 29;4(5):4576–83. doi: 10.1021/acsanm.1c00176 (PMC8165693; doi:10.1021/acsanm.1c00176)
Supplement: Supplementary file 1 — an1c00176_si_001.pdf [file an1c00176_si_001.pdf]

Supporting information

Sensing Enzyme Activation Heat Capacity at the Single-Molecule Level Using Gold-Nanorod-Based Optical Whispering Gallery Modes

Sivaraman Subramanian,[†] Hannah B.L. Jones,[‡,∥] Simona Frustaci,[†] Samuel Winter,[‡] Marc W. van der Kamp,[∣] Vickery L. Arcus,[¶] Christopher R. Pudney[‡], and Frank Vollmer[†].*

†Living Systems Institute, Department of Physics & Astronomy, University of Exeter, Exeter, UK, EX4 4QD. ‡Department of Biology and Biochemistry, Centre for Biosensors, Bioelectronics and Biodevices, University of Bath, Bath, UK, BA2 7AY. ∣School of Biochemistry, University of Bristol, UK, BS8 1TD. ¶Te Aka Mātuatua - School of Science, University of Waikato, Hamilton, NZ, 3240.

Corresponding authors: F.Vollmer@exeter.ac.uk

S1 Sensor assembly

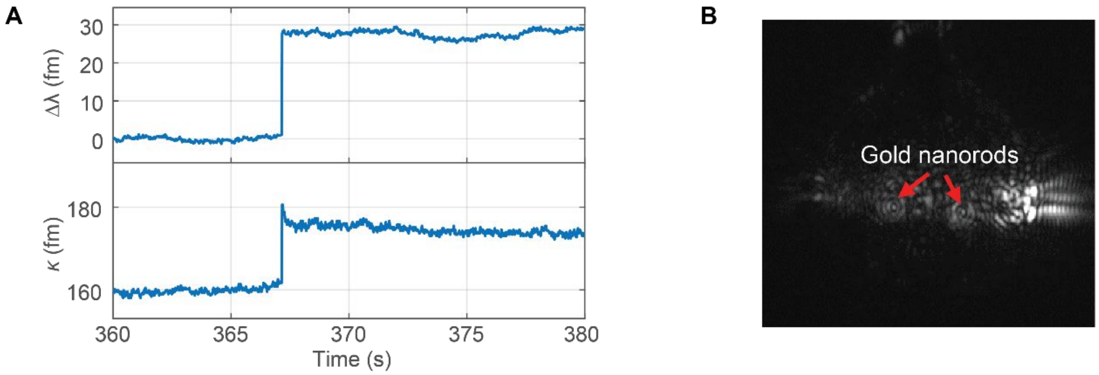

Figure S1. Time traces of the whispering gallery mode (WGM) resonance position Δλ and resonance full-width-at-half-maximum (FWHM) κ. The step transitions indicate binding of single gold nanorods.

As described in the Methods section of the manuscript, the WGM microcavity is fabricated at the end of a single-mode optical fiber (SMF 28e, Corning GmbH, Germany). The glass fiber is first stripped of its protective coating and cleaned with acetone and isopropanol repeatedly to obtain a clean glass fiber. Then, a 30 W CO2 laser, λ ≈ 10.6 μm (Synrad 48-2, Novanta Inc., WA, USA), is used at 10 − 15% peak power to melt the glass fiber into a sphere using a home-built setup. The final diameter of the spherical glass resonator is 80 – 90 μm.

Then, gold nanorods are attached to the surface of the glass resonator. The WGM with plasmonic nanorods attached on its surface acts as the sensor. The gold nanorods are attached electrostatically to the surface of the resonator. Cetrimonium bromide (CTAB) gold nanorods of size approximately 10x35 nm (Nanopartz Inc, USA) are dispersed at a concentration of 500 fM to 1 pM in an aqueous solution of pH ≈ 1.6. Figure S1A shows the step-like transition in the WGM resonance position ($\Delta\lambda$) and full-width-at-half-maximum (FWHM κ) obtained upon the binding of individual gold nanorods. Typically a total of 5-10 nanorods are attached to the surface of the resonator. A photograph of the glass resonator after binding of nanorods is shown in Figure S1B. In the next step, enzymes are attached to the nanorod surface covalently. Multiple enzymes may be attached to the nanorods. Nonetheless, the number of enzymes contributing to the signal can be counted via the step-like transitions as shown in Figure 2B. WGM resonance shifts caused by temperature offsets are not significant for the single-enzyme measurements as temperature effects are much slower (~ s) whereas the single molecule signals are in the timescale of ms.

S2 Pound-Drever-Hall-Locking for high-frequency measurements

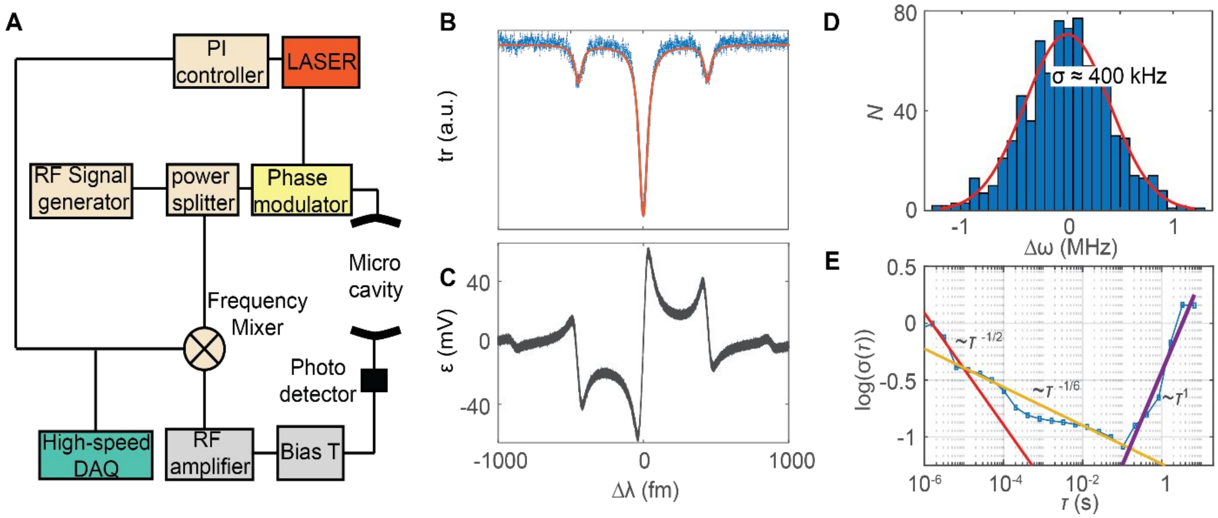

Figure S2. Pound-Drever-Hall lock-in scheme. A, Schematic of the PDH electronics. A RF signal generator drives a phase modulator to produce side bands on the cavity input field. The output is collected with a high-speed photodetector, amplified and mixed with the driving RF signal to produce the error signal. **B,** WGM resonance with side-bands modulated at a frequency of 250 MHz. **C**, PDH error signal obtained after mixing and low-pass filtering the photodiode signal. **D**, Histogram of scaled error signal using an integration time of 5 μs. The standard deviation of the error signal σ ≈ 400 kHz is in the range of the values reported for the laser linewidth (Toptica DL Pro 780, Toptica GmbH, Germany). **E,** Allan variance plot of the WGM resonance frequency measured by the error signal.

The time-resolution of our sensor is ultimately limited by the amount of time light spends in the cavity, that is, the cavity lifetime τ. However, laser scanning using piezo limits the time-resolution of the sensor due to the slow mechanical movement of the piezo stage. Faster tracking of the WGM resonance can be achieved by locking the laser to the WGM resonance. This is achieved by a Pound-Drever-Hall lock-in (PDH) scheme.[1-2] Figure S2A shows the schematics of the electronics to set-up a PDH lock for tracking the WGM. A fast electro-optic modulator (Photline NIR-MPX800-LN-05) is used to modulate the phase of the cavity's incident field at a frequency Ω, such that the incident field takes the form $E_{inc} = E_0 \exp(\omega_0 t + \beta sin\Omega t)$, where $E_0\exp(\omega_0 t)$ is the field before the phase modulation and β is the modulation depth. As a result, sidebands are produced at $\omega \pm \Omega, \omega \pm 2\Omega$, and so on as shown in Figure S2B (top). If the modulation frequency is much higher than the cavity linewidth, then the sidebands do not accumulate any phase information of the cavity. However, the beat note between the carrier and sidebands contains the relative phase information between the incident and cavity fields. This modulated signal is then mixed down using an analog mixer and low pass filtered (Mini Circuits Inc., USA), to extract the derivative signal (also known as the error signal) as shown in Figure S2B (bottom). This

error signal is then proportional to detuning of the laser frequency from the cavity resonance and is given by,[1]

$$\epsilon \approx \sqrt{P_c P_s}\frac{\delta f}{\kappa},$$

where P_c is the power in the carrier, P_s is power in the sidebands and f is the laser frequency and κ is the cavity linewidth. As long as the cavity linewidth is constant, a simple calibration can be used to convert the measured feedback voltages to the frequency shifts. Figure S2C shows the histogram of the frequency shifts measured with the feedback signal of the PDH lock. The standard deviation of the scaled error signal integrated over 5 μs is approximately 400 kHz which reproduces the laser linewidth (Toptica DL Pro 780, Toptica TA Pro, Toptica GmbH, Germany). This shows that the error captures the WGM shift signal and the major noise source is the frequency jitter of the laser. A small electronic noise is added at lower frequencies. Application of improved digital lock-in modules can improve the signal-to-noise removing the electronic source of noise in the future. Figure S2D shows the Allan variance plot of the frequency measured by the error signal after enabling the lock-in. The different slopes of the Allan plot provide information on the possible noise sources. The $\tau^{-1/2}$ slope indicates that white frequency noise dominates in this regime. The

$\tau^{-1/6}$ slope indicates a combination of noise sources, possibly white frequency, and electronic noise. The τ^{1} slope indicates the long term drift. The plot shows that 100 ms integration provides the lowest noise. However, as we require to capture signals in the few millisecond timescales, we use a low pass filter at 100 kHz to acquire the error signal.

S3 Detection of signals peaks and 'event' classification

A graphical user interface is developed in MATLAB (Mathworks Inc, USA) for processing the WGM time traces. The full spectrum of the WGM is obtained using laser scanning is first processed to obtain a WGM resonance position (λ) and linewidth (κ) using a centroid fitting algorithm.[3] A custom Labview (National Instruments Inc., USA) program is used to record and process the raw WGM spectra used to track the WGM resonance position obtained using the PDH lock-in scheme. Once, the WGM time traces are obtained, the data was analyzed for peaks using the MATLAB GUI. First, drift correction is applied to remove the slow variation of the resonance potion ($\Delta\lambda$) if required. The drift of $\Delta\lambda$ occurs due to the slow variation of temperature (on the order $\pm$0.2 K over the course of the experiment) in the sample chamber. In the case of the PDH signal, most of the drift is automatically accounted for by the PID feedback to the laser. However, large amplitude

changes in pressure and temperature are still measured by the error signal. A first-order Savitzky-Golay filter[4] with a window length depending on the sampling rate is applied to the signal.

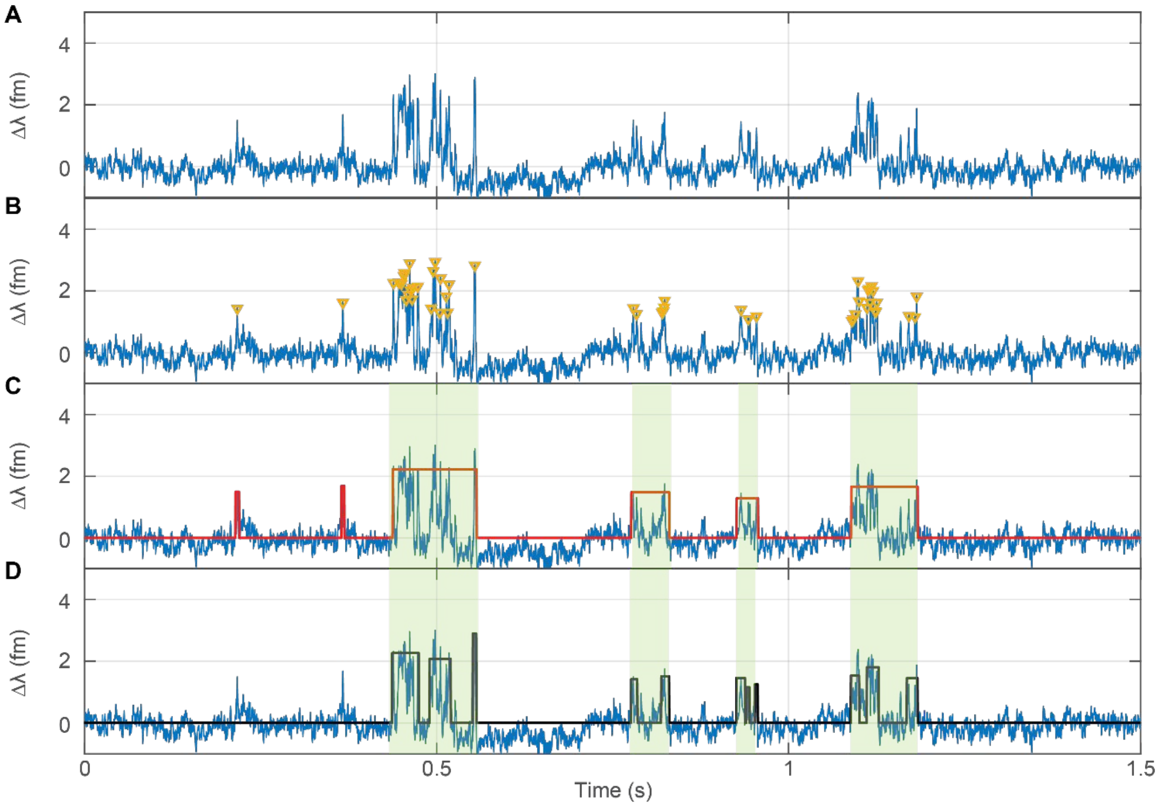

Figure S3. **Peak detection in the WGM time traces. A,** WGM time traces after drift correction. **B,** Individual peaks detected using the peak-detection algorithm. **C,** Grouped peaks (solid line) with a time threshold of 50ms. **D,** Re-grouping peaks to identify subdomains with a smaller grouping threshold of 20 ms.

Figure S3A shows the WGM resonance time traces obtained after drift correction has been applied. Next, a peak detection algorithm based on either the MATLAB function *findpeaks* is used to find the $\Delta\lambda$ values corresponding to single-molecule events. The 'spike' like transitions seen in Figure S3A is defined as the signals. Hence, the signal is similar to the noise but has a higher amplitude. We quantify as signal peaks, all 'spikes' with amplitude higher than $3 - 5\sigma$ (the standard deviation of the background). The value of σ is evaluated by dividing the WGM time trace into windows of N points and evaluating the standard deviation of each N-point window. The minimum value of the standard deviation obtained is taken as the σ of the background. Typically, the value of σ is $0.4 - 0.5$ fm. Then, the threshold of detecting peaks using *findpeaks* is chosen manually between $3 - 5\sigma$. Figure S3B shows the peaks detected in a segment of the WGM time trace. The triangles show all the individual peaks detected above the threshold. The detected peaks are then classified into single-molecule 'events'. A single molecule 'event' defines the set of signal peaks that belong to one set of interactions (one enzyme-substrate interaction in our case). The detected peaks are coalesced into 'events' using the nearest neighbor search based on a grouping threshold. Essentially, any consecutive peaks within a time separation of δt

are coalesced into one single-molecule ‘event’. The grouping threshold δt used in this work is between 50 − 150 ms. Figure S3C shows the peaks detected from Figure S3B grouped into single-molecule events using Δt = 50 ms. Further, single-molecule events can contain sub-domains. These are extracted by using a smaller grouping threshold of 10 − 30 ms (Figure S3D). The signal peaks likely indicate the conformational fluctuations of the enzyme after substrate binding as described in the manuscript. The source code of the GUI can be obtained from https://github.com/ssubram905/WGM_DataAnalysis.git.

Figure S4A plots a long time-trace of the WGM resonance shift measured upon adding 250 μm pNPG after enzyme immobilization at 308 K. The plot has been corrected for small drifts. The individual peaks above the noise (marked by the orange overlay) are detected as described above and grouped into ‘events’. This provides the time of occurrence of the events ‘t’, the dwell time of the events ‘τ’ and the event height ‘h’. Figure S4B plots the survivor function S(Δt) of the time between occurrence of consecutive events ‘Δt’ from the extract occurrence times. The figure also plots the fit to the equation,

$$\Delta A = \sum_{i=1}^{\leq 2} A_i \exp\left(-k_i t\right), \qquad \text{Eq 1}$$

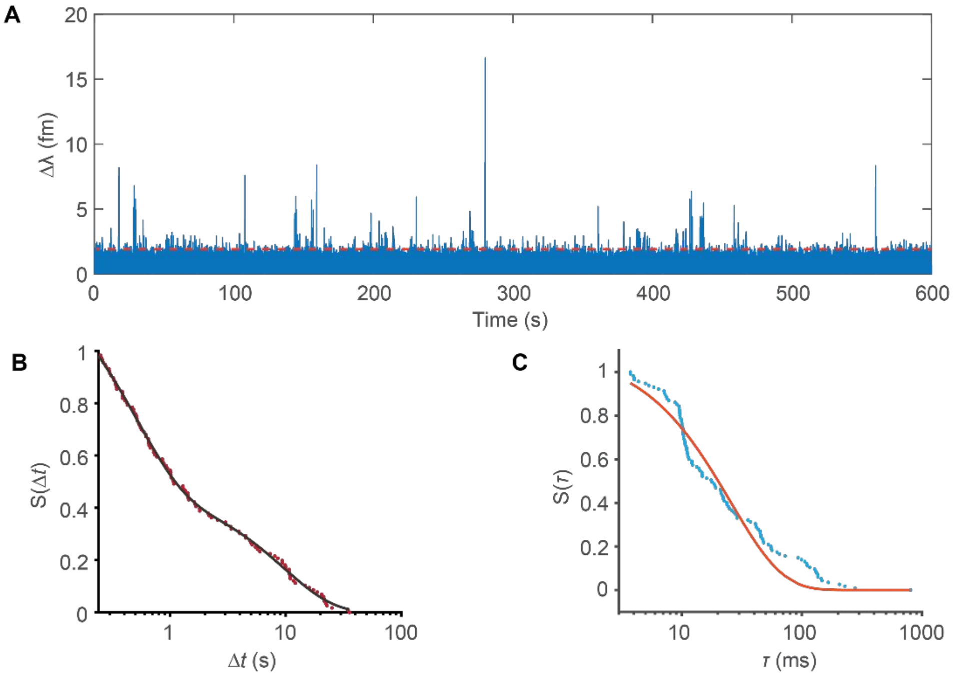

Figure S4. Analysis of the peaks and extracting rates. A, WGM time traces measured upon enzyme turnover after addition of 250 μm pNPG at 308 K after drift correction. The peaks are detected above the threshold (red, dashed) . **B,** Survivor function of the time between events Δt and corresponding biexponential fit to extract rates. **C,** Survivor function of the event duration τ and corresponding single exponential fit to extract the rate.

where A is the amplitude, k is the observed rate constant (k_{obs}) for the ith exponential component (up to two) and ΔA is the total amplitude change. For the Δt data, we use a bi-exponential model as the data deviates significantly from a single exponential fit. The rates obtained from a bi-exponential fit at 308 K are k_1 ~ 120 min^{-1} and k_2 ~ 6 min^{-1}. The rates obtained at all temperatures using the bi-exponential fit are an order of magnitude different. Hence, we surmise that the Δt data are composed of two processes, putatively, as described in Scheme 2. Figure S4C plots the survivor function S(τ) of the event dwell time τ and the corresponding single-exponential fit according to Eq 1. In this case, the data is adequately fit via single exponential and increasing the number of exponentials does not result in a better fit. Increasing the number of exponentials results in rates that are of a similar order and hence a single-exponential model is chosen. The estimated rate k_τ is ~ 2340 min^{-1}.

S4 Colorimetric assays of WT MalL and S135C-MalL

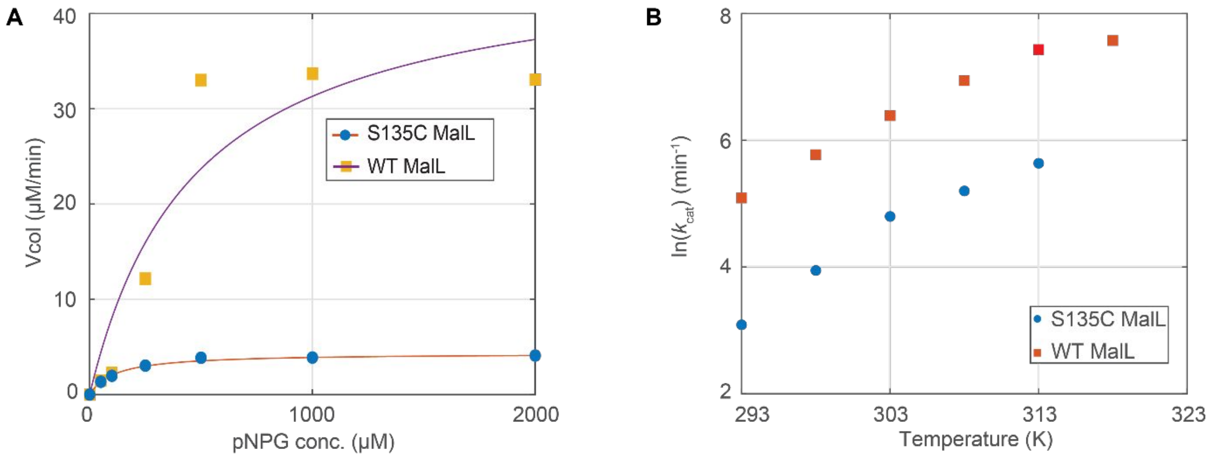

Figure S5. Kinetics of malL measured by colorimetric assay. A, Plots show the Michaelis–Menten kinetics of the WT enzyme and the mutant S135C measured using a colorimetric assay at 308 K at a pH ≈ 7.2 in a 20 mM HEPES buffer (4-(2-hydroxyethyl)1-piperazineethanesulfonic acid). **B,** Plots of the temperature dependent catalytic rate of the WT and mutant S135C measured using a colorimetric assay. The data shows that the mutation has an effect on enzyme activity.

The steady-state kinetics of unmodified wildtype enzyme malL (WT MalL) and the CYS mutant (S135C MalL) were compared using a colorimetric enzyme assay. The enzyme assays were performed using a UV/Vis spectrophotometer (Agilent Cary 60 UV-Vis spectrometer, Santa Clara, CA, USA) in a 1 cm cuvette. The turnover of the substrate PNPG by both enzymes was monitored as the increase in absorption at 420 nm reflecting

product formation. The experiments were performed at pH ≈ 7.2 in a 20 mM HEPES buffer (4-(2-hydroxyethyl)-1-piperazineethanesulfonic acid). The enzyme concentration in the experiments was 100 nM. Additionally, 20 mM TCEP is added to the buffer while performing measurements with the mutant S135C. The initial rates were calculated using an absorption coefficient, $\epsilon = 18\ \mathrm{mM}^{-1}\mathrm{cm}^{-1}$.

Figure S4A shows the steady-state kinetic plots of the two enzymes. The kinetic plots show that the activity of the enzyme is affected by the CYS mutation. The Michaelis constants K_M measured are $K_\mathrm{M} = 107.9 \pm 34.2$ and $141.2 \pm 39.6\ \mu\mathrm{M}$ for the WT and S135C, respectively. Figure S4B shows the temperature dependent turnover rate k_{cat} measured using a colorimetric assay. The value of enzyme catalytic rates k_{cat} are 280.6 min^{-1} and 1688.1 min^{-1} for S135C MalL and WT MalL at 313 K, respectively. The activity of the mutant S135C is approximately 5 fold lower than the WT enzyme at 313 K.

S5 Molecular Dynamics simulation of S135C-MalL

Molecular dynamics simulations are performed using the GROMACS software package.[5] The analysis of the results obtained is performed using GROMACS and VMD.[6-7] The free enzyme simulations are performed using the OPLS[8] force-field and the enzyme-surface

simulations are performed using the GolP-OPLS[9] force-field. The GolP-OPLS force-field includes parameters for amino acid-gold, water-gold, and ion-gold interactions. For the free enzyme simulations a cubic simulation box is used with dimensions 1 nm larger than the enzyme (see Figure S5A). For the enzyme-surface simulations, an Au <111 >surface with 3 gold layers with dimension 102 × 102 × 20 ˚A is created using a unit cell with a lattice constant *a* = 2.93 and unit vectors (1,0,0) and (1⁄2, 3⁄2,0). A cuboidal simulation box with dimensions 102 × 102 × 160 ˚A (see Figure S5B) is used for the enzyme-surface simulations. It is important to note here that the simulation box XY dimension is equal to that of the gold layer so that periodic boundary conditions can be utilized without errors. The enzyme is placed with the 135CYS residue approximately 7.7 Å away from the Au surface (see

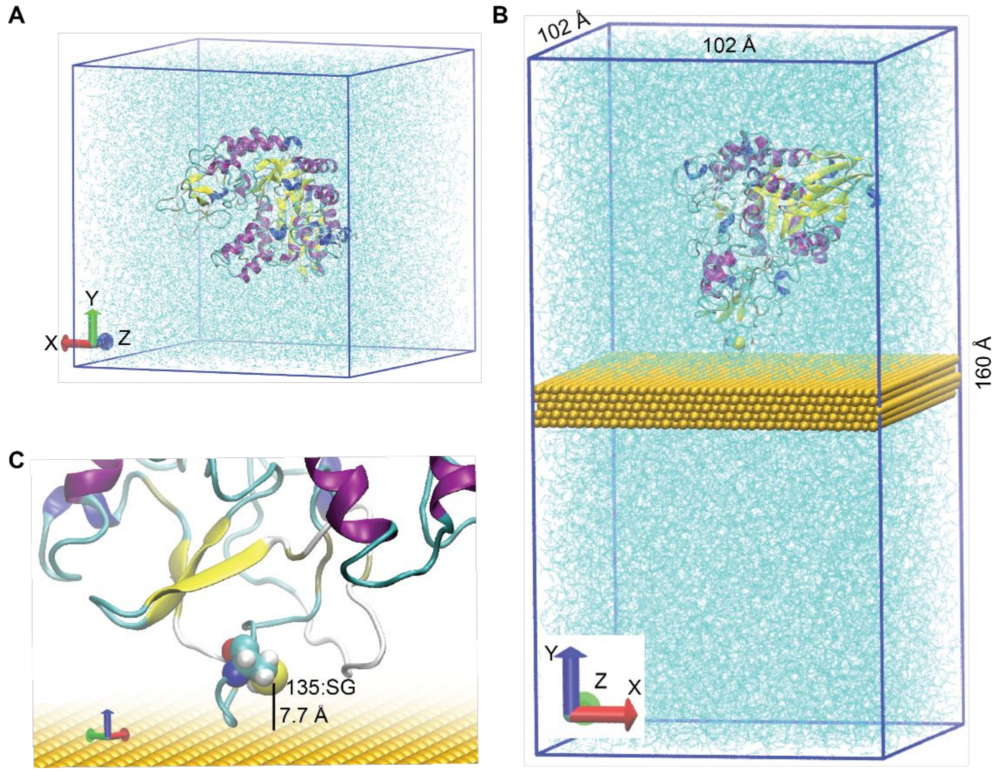

Figure S6. **Molecular dynamics simulations of S135C-MalL. A**, The simulation box of S135C MalL solvated in water as solvent. **B,** Simulation box of S135C-MalL near the surface of an Au $< 111 >$ surface solvated in water. The box dimensions are $102 \times 102 \times 160$ Å. **C,** A zoom in of the starting configuration of S135C MalL on the surface of the gold. The simulation is started with the Sulphur of the residue CYS-135, 7.78 Å away from the gold surface.

Figure S5C). The simulation boxes are then solvated using the SPC/E water model10-11 and compensated for the net negative charge of the system using Na+ ions. The structures

are energy minimized using the steepest descent method until the maximum net force Fmax < 1000 KJ−1mol−1nm−1. After the energy minimization, the systems are temperature equilibrated under the Nose-hoover[12-13] thermostat at 300 K. For the free enzyme system the equilibration is performed in a single step of 50 ps. For the enzyme-surface system the equilibration is performed gradually in 300 steps of 1 K with 50 ps per step. During the equilibration step, the protein is held using a position restraint and the Au surface atoms are frozen. All bonds containing hydrogen are constrained using the LINCS14 algorithm. The production MD runs were performed for both systems in an NVT ensemble at 300 K, with temperature regulated using a Nose-Hoover thermostat for duration of 40 ns. The simulation time-step was 2 fs.

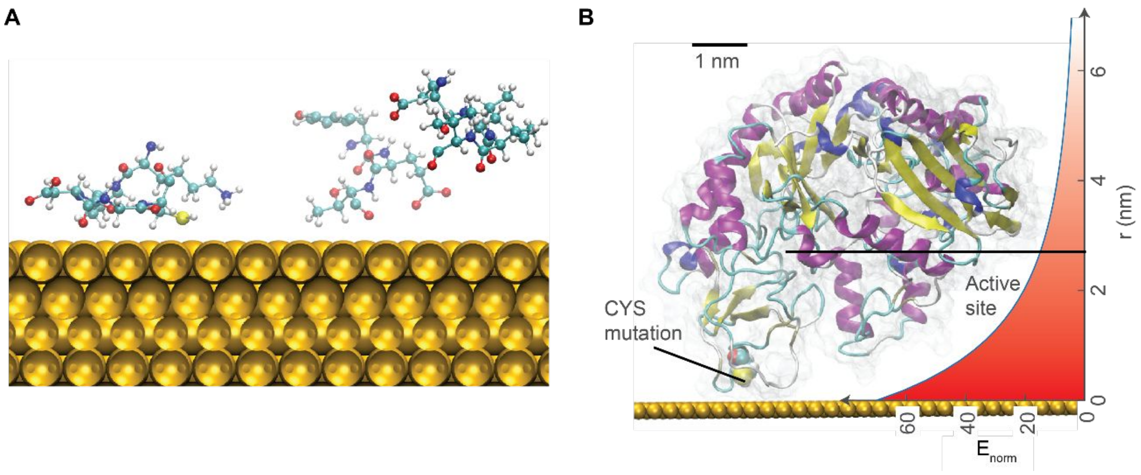

Figure S7. **A,** Representation of the gold surface with the amino acid residues that contact the surface during the simulation. B**,** A representation of the protein on the surface of the gold layer from our simulations. The plot also shows the decay of the near-field intensity of the plasmonic gold nanorod. The active site of the enzyme is approximately 2-3 nm away from the gold surface where the field enhancement is still > 20-fold as compared to the electric-field intensity without the nanorods.

Figure S6A shows a close-up of the gold surface and the amino acid residues of the enzyme that come into contact (defined as residues that are closer than 0.3Å from the gold surface) with the gold surface. The residues 131-135, 213-216, 396-399, and residues 403-404 interact with the surface during the simulation. These residues are mainly composed of LYS, ASP, TYR, and CYS amino acids. Figure S6B shows a representation of the entire

S135C MalL molecule on the surface of the gold layer at approximately 9 ns. The active site of the enzyme is marked and is situated 2-3 nm away from the gold surface. The electric field enhancement provided by the gold nanorod and its decay from the surface simulated using the MNPBEM[15] software is also plotted for the reference. The field enhancement provided by the nanorod is > 20-fold at the site of the active site.

References

(1) Black, E. D., An introduction to Pound–Drever–Hall laser frequency stabilization. *American Journal of Physics* **2001,** *69* (1), 79--87.

(2) Drever, R. W. P.; Hall, J. L.; Kowalski, F. V.; Hough, J.; Ford, G. M.; Munley, A. J.; Ward, H., Laser phase and frequency stabilization using an optical resonator. *Applied Physics B Photophysics and Laser Chemistry* **1983,** *31* (2), 97--105.

(3) Kukanskis, K.; Elkind, J.; Melendez, J.; Murphy, T.; Miller, G.; Garner, H., Detection of DNA Hybridization Using the TISPR-1 Surface Plasmon Resonance Biosensor. *Analytical Biochemistry* **1999,** *274* (1), 7--17.

(4) Savitzky, A.; Golay, M. J. E., Smoothing and Differentiation of Data by Simplified Least Squares Procedures. *Analytical Chemistry* **1964,** *36* (8), 1627--1639.

(5) Abraham, M. J.; Murtola, T.; Schulz, R.; Pll, S.; Smith, J. C.; Hess, B.; Lindahl, E., GROMACS: High performance molecular simulations through multi-level parallelism from laptops to supercomputers. *SoftwareX* **2015,** *1-2*, 19--25.

(6) Humphrey, W.; Dalke, A.; Schulten, K., VMD: Visual molecular dynamics. *Journal of Molecular Graphics* **1996,** *14* (1), 33--38.

(7) Stone, J. E. An Efficient Library for Parallel Ray Tracing And Animation. University of Missouri-Riolla, 1998.

(8) Kaminski, G. A.; Friesner, R. A.; Tirado-Rives, J.; Jorgensen, W. L., Evaluation and reparametrization of the OPLS-AA force field for proteins via comparison with accurate quantum chemical calculations on peptides. *Journal of Physical Chemistry B* **2001,** *105* (28), 6474--6487.

(9) Iori, F.; Di Felice, R.; Molinari, E.; Corni, S., GolP: an atomistic force-field to describe the interaction of proteins with Au(111) surfaces in water. *J Comput Chem* **2009,** *30* (9), 1465-76.

(10) Berendsen, H. J. C.; Grigera, J. R.; Straatsma, T. P., The missing term in effective pair potentials. *The Journal of Physical Chemistry* **1987,** *91* (24), 6269--6271.

(11) Kusalik, P. G.; Svishchev, I. M., The Spatial Structure in Liquid Water. *Science* **1994,** *265* (5176), 1219--1221.

(12) Hoover, W. G., Canonical dynamics: Equilibrium phase-space distributions. *Physical Review A* **1985,** *31* (3), 1695--1697.

(13) Nosé, S., A molecular dynamics method for simulations in the canonical ensemble. *Molecular Physics* **2006,** *52* (2), 255-268.

(14) Hess, B.; Bekker, H.; Berendsen, H. J. C.; Fraaije, J. G. E. M., LINCS: A linear constraint solver for molecular simulations. *Journal of Computational Chemistry* **1997,** *18* (12), 1463-1472.

(15) Hohenester, U.; Trgler, A., MNPBEM – A Matlab toolbox for the simulation of plasmonic nanoparticles. *Computer Physics Communications* **2012,** *183* (2), 370--381.
